# Barriers to Infertility Treatment: An Integrated Study

**DOI:** 10.5539/gjhs.v6n1p181

**Published:** 2013-11-25

**Authors:** Leili Mosalanejad, Nehle Parandavar, Sareh Abdollahifard

**Affiliations:** 1 Department of Mental Health, Jahrom University of Medical Sciences, Jahrom, Iran; 2 Deptartment of Nursing and Midwifery, Jahrom University of Medical Sciences, Jahrom, Iran

**Keywords:** infertility, barrier, infertility treatments, women

## Abstract

**Background:**

Infertility is one of the most important events in life. Despite the negative impact of infertility, a significant number of women struggling to conceive do not consult a physician and do not fallow up infertility treatment. This integrated study aimed to investigate a large amount of factors which influenced discontinuation of infertility treatment.

**Methods:**

This integrated study (qualitative – quantitative study) was done on infertile women who had referred to infertility center in Jahrom University of medical sciences using purposive sampling. In the first study, data were collected from a valid questionnaire with 22 questions in a 5-point likert scale about barriers to infertility treatments and in the second study, as a phenomenology approach, data collection was done using deep unstructured interviews and focused groups were aimed to identify deep individual experiences about it.

**Results:**

major barriers to infertility treatments included the probability of treatment failure (52.5%), couple’s age and possibility of high risk pregnancy (51.5%), Painfulness of some treatment methods such as laparoscopy (50.5%). Qualitative results led to the identification of three main themes: Nature of treatment, negative thinking, social and cultural factors.

**Conclusion:**

As a result, we suggest family education and enrichment of cultural context in the field of infertility; infertile people would be willing to pursue infertility treatments.

## 1. Introduction

Infertility is defined as a lack of pregnancy after one year intercourse without using contraceptives (Merc et al., 2010).

Infertility is propounded as a great social and family problem and its prevalence is estimated to be 10 to 15 percent. Based on previous studies, 12% of Iranian couples suffer from Infertility problems (Sedaghat Siahkal et al., 2003). In contrast, fertility is very valuable in most cultures and is a part of women's power and identity (Abbasi-Shahvazi et al., 2005). Therefore, the desire to have a child is one of the most basic human incentives in life (Fahimi et al., 2010).

Birth of an infant can strengthen family foundation, elevate emotional needs of individuals and finally may lead to renewal and continuation of generation and infertility is a fact that is incompatible with all these cases. Therefore, in an 86- item list of stressful events of life, infertility is one of the most negative events, which amounts to child or spouse death (Sadeghiyan et al., 2006).

Infertility not only creates an important psychological aspect in the individual, but also can act as a powerful hit against the strength of marriage. When patients are deprived of the most important product of their common life, they not only will lose the meaning of their life, but also will lose the meaning of their intermediate relations.

Close intimacy and affection will be lost in cases that couples need it more than anything (Zabihi et al., 2012). Concern and fear of how to expose this problem with one’s husband and also fear of collapse of family life are the first horrors that force a woman to take an action for releasing from treatment deadlock in any possible way. the love of becoming a mother and patriarchal beliefs for surviving and continuing the generation, a lack of social and economic support for most women, low chance of remarriage for an infertile woman and on the other hand, blaming single life in Iranian culture, are among the factors which redouble the problems caused by infertility for women (Fahimi et al., 2010). Fortunately, today, with the advancement of technology, more than 90% of diagnosed infertility cases succeed and available evidence shows that, less people will be deprived of having a child (Quaas et al., 2008).

The ratio of couples who followed medical treatments has been reported to be 56.1% (about 42-76.3%) in developed counties and 51.2% (about 27-74.1%) in developing countries (Boivin et al., 2007).

But what introduces infertility as a stressful factor is the problems of infertility treatment that causes discomfort, complications and failure of medical methods for women (Bakhshayesh et al., 2012).

In addition to mental, family and social problems, this group suffers from economic problems (the costs of referring to several physicians and treatment centers, tests, etc) that is naturally stressful (Ryan Kenneth et al., 1999). High cost of assisted reproductive techniques and a lack of insurance coverage by insurance institutes are among major problems of infertile couples (Roni Berger et al., 2013).

Existing barriers and problems in the treatment procedure causes infertility to be considered as a kind of life crisis, a chronic disease, a harm and a cause of distress in these people (Diamond et al., 1999).

Existing problems in the treatment procedure especially cause the appearance of impulsive behaviors, scattered wrath, depression, feeling of helplessness, worthlessness, incompetence, anxiety and negative beliefs towards themselves within and after long-term and sometimes unsuccessful treatments (Burns, 2007). Sometimes these stresses, helplessness and mental problems can underlie the discontinuation of treatment.

In a systematic review of 22 studies carried out by Gameiro et al. (2012) reasons of treatment discontinuation included:

Treatment delay (39.18%), unknown cause (19.17%), physical and mental burden (19.07%), mental burden (14%), physical burden (6.32%), personal problems (16.67%), personal reasons (9.27%), communication problems (8.83%), refusal of treatment (13.23%), organizational (11.68%) and clinic (7.71%).

Knowing the barriers to infertility treatments, especially from the viewpoint of the people involved in this procedure, can lead to present appropriate solutions in the field of infertility treatments and can also play an important role in designing treatment programs for infertile couples (Zabihi et al., 2012). This article aimed to evaluate infertile women's experiences of infertility treatment barriers with phenomenological approach and to discover barriers to infertility treatment in this group of society.

## 2. Materials and Methods

This study is a mixed method of research by triangulation method, which involves the use of both quantitative and qualitative approaches. This research has been carried out on infertile women who had referred to Jahrom maternity clinics in 2012-2013. Using between-method triangulation was recommended by Denzin (1978), by putting emphasis on utilizing mixed methods “the inherent bias in any particular data source, investigators, and particularly method will be canceled out when used in conjunction with other data sources, investigators, and methods”.

following advantages of triangulation were noted by Jick (1979): (a) it allows researchers to be more confident of their results; (b) it stimulates the development of creative ways of data collection; (c) it can lead to thicker, richer data; (d) it can lead to the synthesis or integration of theories; (e) it can uncover contradictions, and (f) by virtue of its comprehensiveness, it may

Be served as the litmus test for competing theories.

Also we used sequential triangulation in the study design. According to Morse (1991), sequential triangulation is utilized when the results of one approach are necessary for planning the next method.

In quantitative part, a researcher-made questionnaire with 22 questions was used in a 5-point scale (very much-very little) on 100 women referred to infertility center.

Its validity was confirmed by 10 specialists in the field of obstetrics and gynecology and its reliability was calculated using Cronbach’s alpha for surveying the internal consistency of the questions on a group of 30 persons using pilot study. The response rate was 83%. The barriers of infertility were extensively surveyed by a quantitative research which was carried out on 100 people who had received assisted reproductive treatments.

In qualitative parts (as a phenomenology study), subjects were included in the study by purposive sampling method from 32 women to whom assisted reproductive treatment had been recommended. "Phenomenological description is a method of approaching phenomena within the area of phenomenology that attempts to understand the structure of lived experience rather than explain it" (Streubert & Carpenter, 2005).

Inclusion criteria included: Persian speaking people referring to maternity clinic because of infertility treatment and discontinuation of the treatment or lack of treatment and follow-up procedure due to different reasons during the treatment. None of the individuals had chronic physical and mental diseases.

These individuals had primary infertility and participated in the interview. Purposive sampling method and semi-structured interview were carried out. If permitted by participants, dialogues were recorded; otherwise, they were written word by word.

The main question of the research was about barriers to infertility treatment and reasons for not following the treatment.

After collecting the information, interview texts were studied for several times and key parts were distinguished and specified in form of conceptual codes or direct quotation (similar to what the participants had said). Similar codes were placed beside each other and classified through their similar with each other and then, main themes were identified.

### 2.1 Data Analysis

Collaizzi seven-stage method used for analyzing the data in this research. All the conversations were recorded, if not, they were written word by word. The main question of this study was the barriers to infertility treatment and related factors.

These processes included:

Reading and re-reading descriptions,

Referring to narratives and extracting significant statements.

Formulating the meanings

Categorizing into clusters of themes and validating them.

Describing

Returning (collaizzi, 1978).

The rigor of such research includes “credibility”, “transferability”, “dependability”, and “conformability” which are determined in the present research. In order to promote the rigor of research, in addition to the main researcher, other members of the team (at least 2 people) actively participated in all stages of analyzing and explaining the codes. Also, the researchers provided a report of all research stages including data collection, data analysis, results and different steps of the research procedure, and gave them to two experienced professors in the field of qualitative research. The codes were used after they were reviewed and agreed on.

The records of all research stages also provided the possibility of its transitivity to researchers. Ethical considerations in this research were: satisfaction of participants in interviews, permission to record interviews and effectiveness of their treatment procedure. Participants’ privacy and comfort was provided as well.

Personal characteristics of participants were kept confidential and they were assured that the audio file will be deleted after use.

## 3. Results

According to quantitative results, 81.1% of individuals were in age range of 21-30 years. Most individuals had diploma (63.4%) and 71.5% reported infertility history of about 5-7 years. 57.5% had experienced assisted reproductive treatments for the first time. The frequency distribution of treatment barriers showed that the highest percentage is related to the probability of failure of treatment (52.5%), couple’s age and possibility of high risk pregnancy (51.5%) and then Painfulness of some treatment methods such as laparoscopy (50.5%).

**Table 1:** Descriptive data from barrier of infertility treatments

	Factors	Too much	Much	Moderate	Little	Very little
1	High cost of infertility treatment	7.45%	10.3%	21.8%	24.5%	36%
2	Lack of financial ability for paying treatment costs.	11.1%	15.2%	18.2%	28.3%	27.2%
3	Lack of couple’s mental preparation for caring out the treatment.	44.1%	21.3%	27.5%	3.6%	3.5%
4	Believing in possibility of natural pregnancy because of low age of woman.	31.6%	24.5%	16.3%	15.3%	12.3%
5	Lack of husband’s tendency to follow up treatment for receiving gametes and embryo from strange persons.	25.3%	12.5%	11.3%	12.2%	38.7%
6	Husband’s disagreement for receiving gametes and embryo from strange persons.	28.2%	25.4%	18.6%	13.3%	14.5%
7	Lack of couple’s complete awareness of new methods of infertility treatment such as, laparoscopy, IUI, artificial, insemination, surrogacy and receiving gamete from the other person.	9.3%	16.4%	15.7%	22.3%	.36.3%
8	No taking prescribed drugs properly and timely in order to treat infertility.	24.3%	18.5%	23.3%	14.9%	19%
9	Man’s healthiness and existence of female-related cause of infertility.	39.8%	16.3%	25.5%	11.2%	7.2%
10	High age of the couples and possibility of high risk pregnancy.	51.5%	14.1%	17.2%	9.1%	8.1%
11	Lack of infertility treatment center in city of residence and problems of coming and going.	42.3%	32%	8.2%	12.4%	5.1%
12	Lack of couple’s family for following up the treatment matters.	17%	15%	18%	19%	31%
13	Having stress about relative’s and acquaintance’s awareness of referring for treating infertility.	39.8%	16.3%	18.4%	9.2%	16.3%
14	Existence of religious problems in some methods of treatment such as surrogacy and receiving gamete from strange persons.	19.2%	18.7%	23.2%	14.3%	25.6%
15	Lack of acceptance of new treatments of fertility from relatives and society such as IVF, surrogacy and receiving gamete.	24.2%	22.2%	13.1%	18.2%	22.3%
16	Prolonged period of infertility treatment.	12.5%	14.4%	13.7%	5.1%	54.3%
17	Medical (treatment) staff’s improper behavior.	25%	17%	20%	18%	20%
18	Lack of giving proper education to couples by medical staffs about the importance of taking drugs properly and timely.	28.3%	23.2%	%22.2	%8.1	%18.2
19	The probability of failure of treatment.	52.5%	21.3%	12.2%	6.1%	7.9%
20	The stress caused by probable failure of treatment method	28.3%	23.2%	22.2%	8.1%	18.2%
21	Painfulness of some treatment methods such as laparoscopy	50.5%	22.2%	13.1%	5.1%	9.1%
22	The complications of used medications in order to prepare for treatment, such as fatness (obesity) and hirsutism.	44.2%	12.6%	16.8%	12.6%	13.8%

There were positive relationship between infertility causes and Painfulness of some treatment methods (p=0.03), failure of treatment method (p=0.03), and the complications of used medications (p=0.02). Also there were positive relationship between educational levels and Painfulness of some treatment methods (p=0.04), high age of the couples and possibility of high risk pregnancy (p=0. 04) and high cost of infertility treatment (0.03).

In the qualitative part of the study which aimed to survey barriers to infertility treatment, 78 codes were extracted from interviewing 32 infertile women. The results showed that the average age of women was 29±2.6. The majority of infertile individuals (76.4%) had female causes (or factors) of infertility, others (14.3%) had male causes of infertility while 11.3% had unknown causes. The average treatment duration in most cases was 8±1.4. 56.3% of individuals had at least experienced treatments with IUI and 16.3% had experienced IVF. Other individuals were using drug treatment for solving infertility problems. All of these individuals had regularly experienced the history of discontinuation of treatment procedure or a lack of follow – up treatment. Results led to identify 3 main contents as follows:

The nature of treatment (physical complications, failure of treatment procedure, centralized facilities and prolonged treatment procedure), negative thoughts (uncertain future, failure of the treatment [lack of success and good results], mental filter) and social & cultural factors in the environment with sub- contents of (traditional and superstitious viewpoint and cultural sensitivity to fertility).

**Table 2:** Themes and sub-theme from barrier of infertility treatments

Sub-themes	Themes
Physical complications	**Nature of the treatments**
Low success of the treatments	
Centralized possibilities	
Prolonged treatment procedures	
Uncertain future	**Negative thoughts**
Failure to get results	
Mental filter	
Traditional and superstitious viewpoint	**Social and cultural factors governing the environment**
Sensitivity to fertility	

The first identified content is the nature of infertility treatment which can be consisted of major factors such as physical complications resulting from infertility treatment, low success or failure of treatment procedures, existence of centralized facilities in provincial centers, states of fear and hope following prolonged procedure of treatment which takes time, going through long distances and finally, failure of treatment procedure and the need to repeat the treatment.

Some of the individuals indicated physical problems resulting from treatment as follows:

"I had IUI treatment 3-4 months ago; the medications which I was taking had side effects. Due to allergy, nausea, vomiting, headache and vertigo, the treatment was so difficult for me. Then, doctors said that: “you should be treated with IUI” and then they performed it."

Another person states that *: "I take drugs, now I came for ultrasound; my drugs are Letrozole, Tamoxan, estrogen and metformin. Metformin causes me to have bad feelings, but I take it, complication and side effects of drugs are a lot, but I take them for having a baby."*

High cost of treatment is one of the most important barriers to infertility treatment and is seen in all stated experiences. The individuals, who a had good financial ability, also complained about high costs of treatment, lack of insurance payments and even supplementary insurance and consider them as the most important factors in giving up the treatment since they complained about not having enough money for their water and electricity bills, and that nothing would be left for drug and doctor."

"I decrease living costs; I buy less food and clothing. We monthly spend 4000-5000 thousand Rials for treatment and we don’t save any. We even delay the payment of bills."

The distance and lack of facilities in small centers have been stated as one of the other problems. On the other hand, lack of infertility centers, shortage of skillful physicians and going through long distances also force individuals to stop and discontinue the treatment procedure.

One of these women states the problem as follows:

"High costs of the treatments, fear of the possibility of failure and that I was going to Shiraz for about 6 months, but the distance was far away and also I could not endure coming and going. So, I stayed here with the specialists who are here. Because I don't want to go to Shiraz for a visit."

A 28-year old woman with 10 years of infertility history says that:

"I have been treated for 9 years, I also went to Tehran, Isfahan and Shiraz for treating, I have been coming and going for 9 years, my treatment has been prolonged and I haven’t gotten a result, the costs are also a lot so that make one disappointed."

### 3.1 Negative Thoughts

Resulting from the problems of infertility treatment, the image of a vague future in life and fear of treatment failure are some of the negative thoughts of infertile individuals that extend negative thoughts and feelings as a mental filter and provide a negative image of future, treatment and its process.

A woman with 13 years of infertility history stated that:

“I'm feeling very bad for not getting pregnant. I become very sad, I'm irritable but I'm not disappointed, I blame myself but I say that I become a housewife, I will have a child too, but I became a low weight and low appetite person."

Another woman stated that:

*“I have negative thoughts. I'm afraid that people tell me you are not pregnant”*.

Negative thoughts and feelings have been stated by one of the women. This woman who also suffered from spouse abuse stated that: "I'm afraid of menstruation, my husband hits me during menstruation as if his father has died, I feel stress, I'm sleepless, when my menstruation time comes, my husband begins to abuse and before it, bad thought comes to my mind, what should I do this time that I am menstruating?"

Another person stated the problems of her negative thoughts as follows:

*“Because I always have irregular menstruation, I'm afraid not to get pregnant. I compare myself with others*. *Recently, my sister-in-law has given birth to a baby. It is very difficult and unpleasant for me to see others who can have a baby, but I can't. I think that I'm not a good daughter-in-law for my husband's family. I am tender-hearted. My heart breaks easily and negative thoughts often come to my mind"*.

Negative thoughts and creating a mental filter which transmits negative mental thoughts in mind are observed in experiences of some women.

These negative thoughts only hurt individuals and their feelings as a corrosive factor of soul and mind.

### 3.2 Socio-Cultural Factors

The style of individual's thought (infertility is only specific to women), customs (having tendency to common treatments with herbal medicine or waiting for the occurrence of natural fertility), cultural sensitivity towards fertility and early interventions, are important considering social and cultural values and causes that, the problems resulting from infertility appear heavier on their families.

A woman who cannot get pregnant because of her husband's problem stated that:

*“The cause of our dispute is not having a baby. I referred to doctor and she said that I didn’t have any problems; your husband should be tested. Even, once I referred to doctor with my brother-in-law and the doctor told him, too. But my husband did not agree. He says: "If God wills, we will have a baby"*.

Refusing to accept a new treatment is another problem that has provided the field of non-acceptance of infertility treatment and intervention in some individuals from different cultural and social dimensions.

"My husband says that, it is better to implant a baby, but I don't want to implant a baby by a device. Because a one-month baby is implanted in my abdomen. I don't want to be like this. I want that the sperm be in my body. This is a good suggestion, but our culture doesn't accept it."

Sensitivity to fertility is seen in the majority of Iranian families. Tendency to have a child has been internalized in Iranian family culture and especially is seen in small cultures more and more.

Regarding family’s sensitivity to fertility and society's emphasis on pregnancy, a person stated that:

"The first treatment barriers are stress and people's culture. Problems such as the sensitivity that you should give birth to a baby soon. The culture of people is so that they don't understand and tell superstitious things."

Hearing others' talks, sarcastic words and inappropriate behavior of some individuals can provide the field for concern and discomfort for some people.

Someone reproaches "when I hear these words, I get really upset and my husband says that: others have children but we don’t have any."

*"Some people know that we don't have a child. But they ask us: don't you have a baby? My husband gets upset and then I begin to cry following him. Everyone says that, by passing the time your disease become chronic. Even when I went to a party and see people with their children, I get really upset. Everyone tells me: what do you do? Why didn't you get pregnant?*"

## 4. Discussion

This study was aimed to survey treatment barriers among infertile couples. Based on our results, treatment cost is the most important barrier even for high income people. Mental problems and feelings are other major factors that influence the treatment procedure.

One of the expectations from forming a family is to have children; this can strengthen the structure of the family. Also, it can supply individuals' mental and emotional needs and lead to continuation of the generation. It is natural that, individuals' social place will be lost by losing this desire, and they will have various mental and emotional problems in family and society such as fear of being rejected, separation, divorce and inconsistency of marriage (Abbasi-Shahvazi et al., 2005).

Humiliation, disappointment, isolationism, and obsession are among the feelings that have been propounded by study participants; the results of Saghfi et al’s study also confirm the appearance of these problems during treatment (Saghafi, 2004). Another study also has shown that one third of infertile women have mental problems and 10% of them have different degrees of depression ranging from moderate to severe (Lok et al., 2002).

Most infertile women believe that, they cannot tolerate life without a child and since most treatments are for women, thus, they are exposed to more damage and risks in comparison with men (Audu., 2002). It should be emphasized that, infertile couples also need mental treatments and support in addition to physical treatments; considering mental needs is one of the necessary and important parts of infertility treatments (VA., 2002).

Oliviou et al’s study has shown that, 242 couples who had discontinued treatment procedures by IVF, stated the cause of treatment procedure discontinuation as follows:

Mental burden (26%), weak prognosis of treatment (25%), spontaneous pregnancy (19%), physical stress (6%), serious disease (2%) and other causes (7%). In majority of these individuals, discontinuation of treatment was due to mental stress. Although stress has not been indicated as a barrier to treatment procedure in this study, but this factor is sensed by each reader in the depth of the discussions (Olivius et al., 2002).

Also, Brende’s study showed that major reasons of discontinuation of treatment by IVF include mental and affective problems (22.3%), weak prognosis of treatment (18.8%) and unsuccessful treatment (17.2%). The rate of spontaneous pregnancy after treatment discontinuation was reported to be 10% (Brandes et al., 2009).

It has been shown in this study that, one third of the individuals discontinued treatment procedure before any infertility treatment; major reasons for discontinuation were affective distress and weak prognosis of treatment. These results indicate couples' need to carry out correct and proper consultations before the initiation of treatment and also infertile couples' sever need to mental supports from centers that provide such services. Also, the present indicated failure in getting results, a vague future and negative thoughts. Moreover, as mentioned above, infertile women who had failure in treatment procedure may suffer stress and distress and sometimes this feeling leads to discontinuation of treatment.

The results of Domar’s study also confirm our results and count depression and anxiety among the results of previous treatment failure experiences in infertile women as causes of treatment discontinuation (Domar et al., 2005).

In this study, the most common reason for discontinuing the treatment is stress (39%) and the main cause of this stress is the loss due to infertility treatment or in the other words the fear of treatment failure that affects couples' relationships and makes them too much anxious and depressed (Domar et al., 2005).

Lack of infertility centers, shortage of skillful physicians and long distances are among mentioned barriers in the present research that force individuals to discontinue the treatment. The results of Fahami's study also indicated existing challenges in treatment procedure such as inappropriate functioning of the treatment team (Fahimi et al., 2010).

When pregnancy does not occur, sexual relations in one's mind changes into futile sexual relations and decreases the libido which follows it. Couples gradually forget that their sexual relation is also a response to their natural need (Mohammadi et al., 2001).

In confirmation of the results of the present research, findings of Abbasi-Shovazi’s research also showed that the most important factor which caused women to suffer from infertility is the fear of losing their common life (Abbasi-Shahvazi et al., 2005).

This fear is clearly specified in the context of interviewees on one hand, which is indicative of created negative thoughts in infertile women and on the other hand, it is indicative of ruling cultural-social status which shows that, failure to give birth to a child will be shaken in this society. Finally, the grief of not having a child causes couples to have various mental problems, which is potentially stressful. Therefore, coping with stress is an important and essential issue. Although different variables are effective on how to respond to stress, but the individuals' values and beliefs are also effective on the way they assess events and encounter with them (Mattlin et al., 1990). Previous studies showed that to what extent variables such as age, job, education and personality characteristics are effective on how to respond to stress and also using coping methods by infertile couples. Use of different coping methods has different results in individuals’ mental health (Esther et al., 2006; Callan and JF., 1989).

Some studies have shown that, because individuals with mental health and relaxation experience less mental stress, there is more possibility of fertility for them (Domar et al., 2005).

Findings of Sedighi’s et al research showed that infertile women's anxiety has decreased after preparation interventions at pre-treatment stage in comparison with the beginning of the research (Sadeghiyan et al., 2006).

It is believed that, due to positive effects of preparation it can be possible to use preparation in pre-treatment programs for decreasing anxiety and consequently increasing the rate of women's fertility.

On the other hand, because of the existence of different causes in failure of infertile couples' treatment procedures, the use of some interventions can decrease exiting barriers to treatment procedure, interventions such as:

Providing psychological counseling in infertility treatment centers at primary stages of treatment for each couple and also their parents as supporters, providing necessary and proper awareness about infertility and treatment procedures and also supportive systems exiting in each system (Sedighi et al., 2005).

It seems that, most preparations should be toward changing couples' attitude and even in broader dimensions, changing society's attitude towards infertility. In a research which aimed to survey treatment discontinuation causes in infertile individuals receiving infertility treatments, mental barriers were the most common causes of treatment discontinuation, and financial barriers had the lowest average followed by physical barriers and individuals' age were among other effective factors. Lack of expertise of treatment staff, negative effects of infertility on individuals' social dimensions and treatment costs had the lowest rate (Van den Broeck et al., 2009). Also, in the present research, person's age and fear of high risk pregnancy had the highest rate and treatment costs had less severity compared with other barriers. In a research carried out on 384 infertile couples, the rate of treatment discontinuation was 17% and mental and physical barriers had the highest frequency (28%). Whereas, the use of treatment strategies even ovum stimulation didn't have any significant effect on treatment discontinuation. But, when other treatments such as IVF were used, the rate of discontinuation of treatment was more than 50% along with anxiety. Male infertility and failure of the treatment procedure were mentioned as other factors of treatment discontinuation (Verberg et al., 2008).

Others showed that, lack of agreement about treatment and related procedures is not a reason for discontinuation of treatment (Gameiro et al., 2012). In another research, two important problems were indicated as well, treatment costs and the important role of stress in failure of the treatment procedure. it is emphasized that the role of stress and costs especially for drugs and medical treatments should be surveyed and consulted before the beginning of treatment (Malcolm and DC., 2006).

The results of the present research also showed that couples' stress due to increasing age, painful treatment procedures and treatment costs had a higher average. In another research, discontinuation treatment was classified to three parts: patient’s problems, treatment problems and clinic-related factors. Therefore, it is necessary to use comprehensive educational materials, screening for identifying patients with severe anxiety, providing the design of coping tools and improving clinical environment in medical interventions in order to reduce the burden of treatment (Van Dongen et al., 2010). This research has indicated different factors that cover these areas in both quantitative and qualitative parts. In another research, natural pregnancy, specified medical or social reasons (for example, separation) and immigration were prevailing reasons for discontinuing the treatment.

Also, women who had been included in the treatment procedure because of their husband's oligospermia, experienced high rate of natural pregnancy. Age, social and economic status, geographical condition and infertility duration did not have any effect on discontinuation of treatment (Pénélope et al., 2012).

In another research most women were delaying treatments because of having the tendency to get pregnant naturally. 35+ year-old women were spending more time for following up treatment procedure. Anxiety was also the second and the greatest barrier to treatment procedure. Also, in the present research, negative thoughts and anxiety resulting from it are seen in different dimensions of the treatment. The other mentioned factor was treatment costs and related psychological factors that were along with physical barriers and clinical reasons. The results of the previous studies are consistent with some obtained results in the present research (Brennan et al., 2006).
